# Supplementary material for: Significant Genes Associated with Mortality and Disease Progression in Grade II and III Glioma
Source: Biomedicines. 2024 Apr 12;12(4):858. doi: 10.3390/biomedicines12040858 (PMC11048596; doi:10.3390/biomedicines12040858)
Supplement: Supplementary file 1 [file biomedicines-12-00858-s001.zip › Supplementary Material.pdf]

Supplementary Materials

Significant genes associated with mortality and disease progression in grade II and III glioma

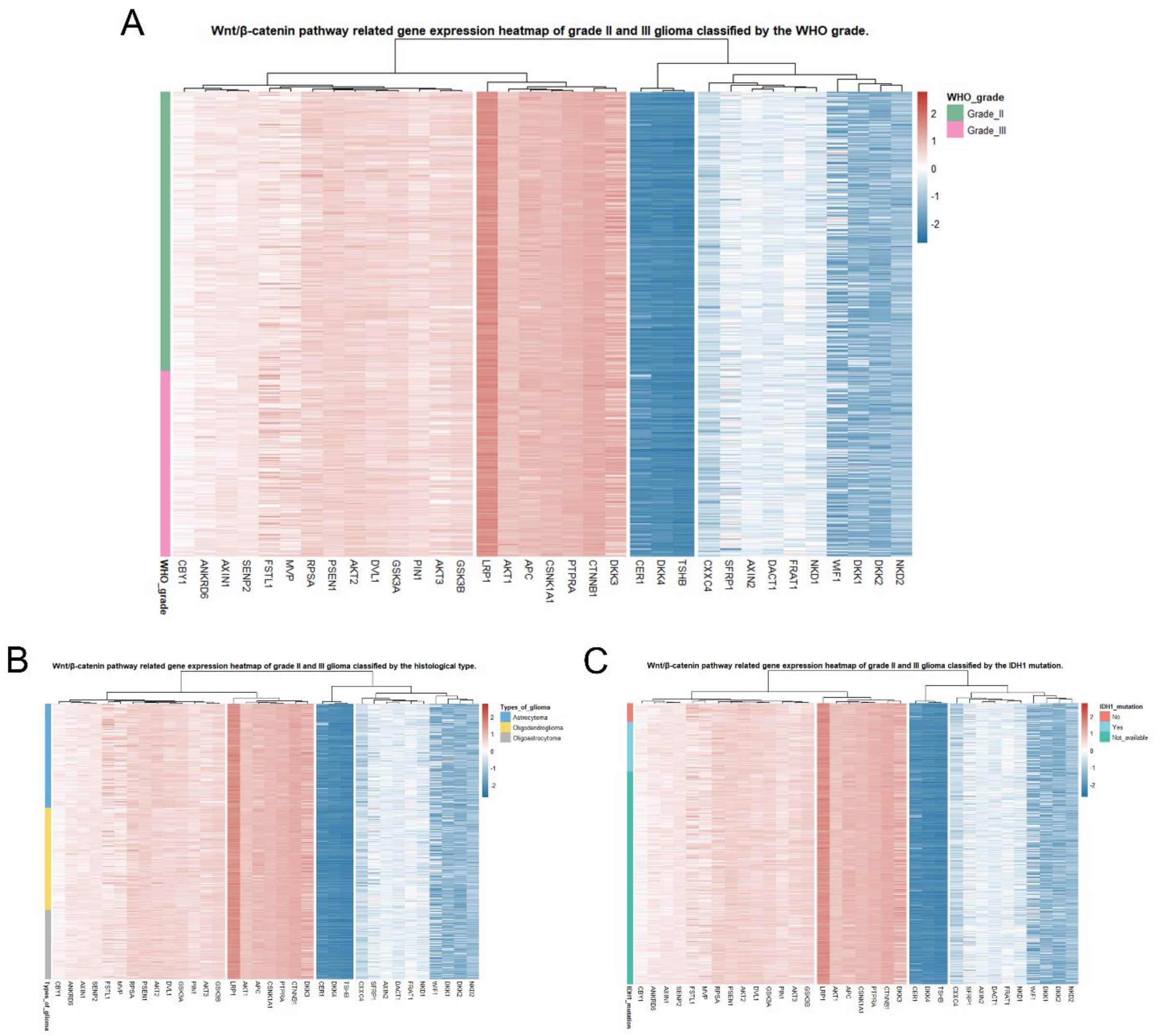

Supplementary Figure S1. Heatmaps showing the expression patterns of 34 genes related to Wnt/β-catenin signaling pathway in patients with grade II and III glioma. Gene expressions were log2 transformed and color density indicating levels of log2 fold changes are displayed. Red and blue represent up- and downregulated expression in grade II and III glioma, respectively: (A) Heatmap showing the expression patterns of 34 genes related to Wnt/β-catenin signaling pathway in patients with grade II and III glioma classified by WHO grade; (B) heatmap showing the expression patterns of 34 genes related to Wnt/β-catenin signaling pathway in patients with grade II and III glioma classified by histological types of glioma; and (C) heatmap showing the

expression patterns of 34 genes related to Wnt/β-catenin signaling pathway in patients with grade II and III glioma classified by *IDH1* mutation status.

WHO, World Health Organization; IDH, isocitrate dehydrogenase.

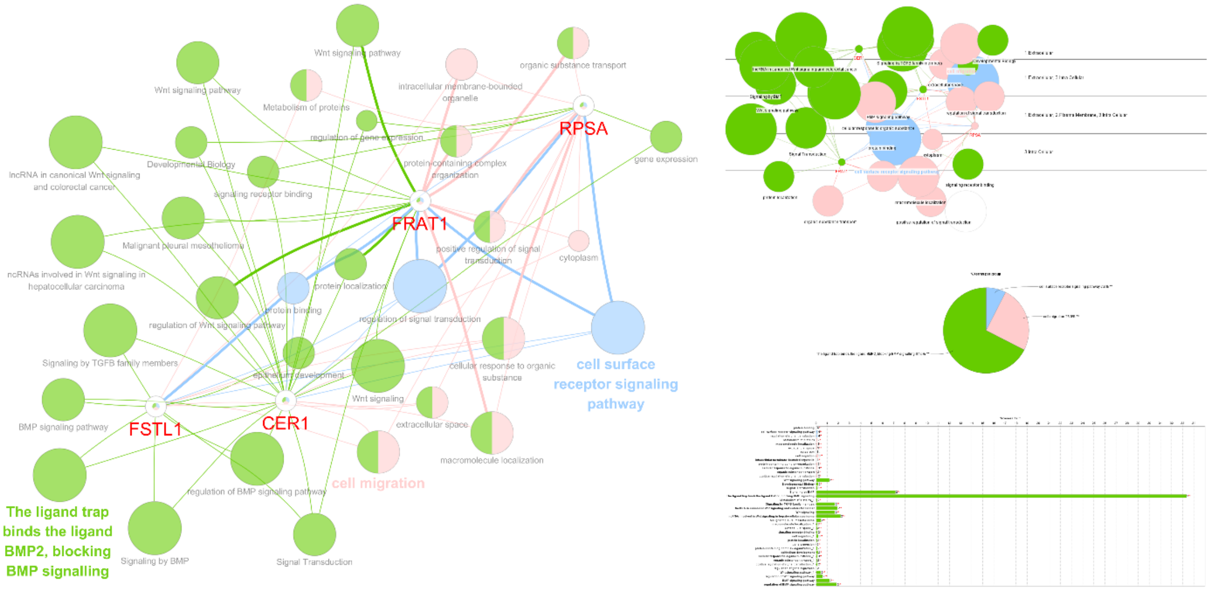

Supplementary Figure S2. Bioinformatics network analysis visualizes the interactions and subcellular localization between four significant genes identified in this study (*CER1*, *FRAT1*, *FSTL1*, and *RPSA*). The network was generated using Cytoscape with functionally enriched GO terms and biological pathways.

GO, gene ontology.

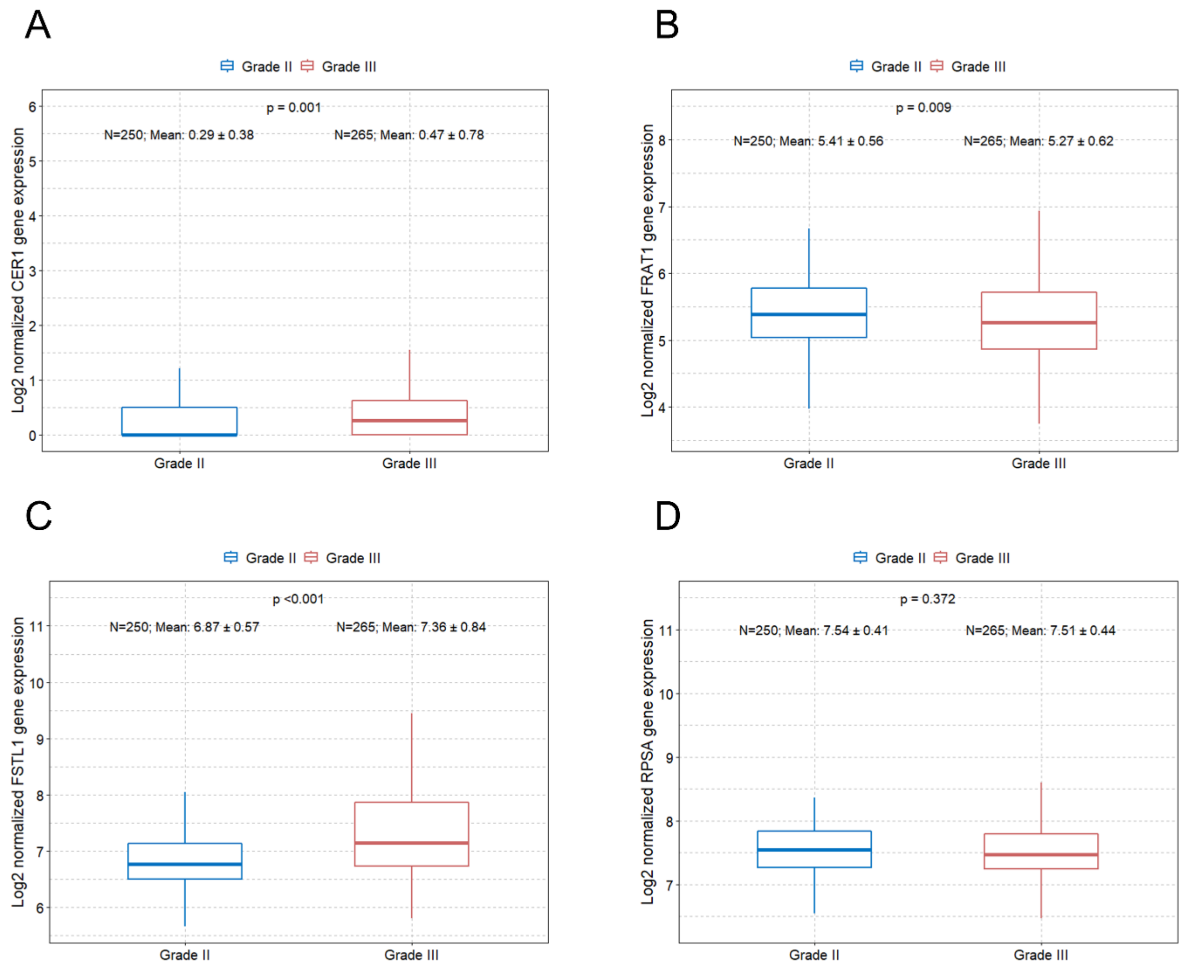

Supplementary Figure S3. Comparison of gene mRNA expressions between grade II and grade III glioma based on four significant genes related to Wnt/β-catenin signaling pathway: (A) *CER1*; (B) *FRAT1*; (C) *FSTL1*; and (D) *RPSA*.

CER1, cerberus 1; *FRAT1*, FRAT regulator of WNT signaling pathway 1; *FSTL1*, follistatin like 1; *RPSA*, ribosomal protein SA.

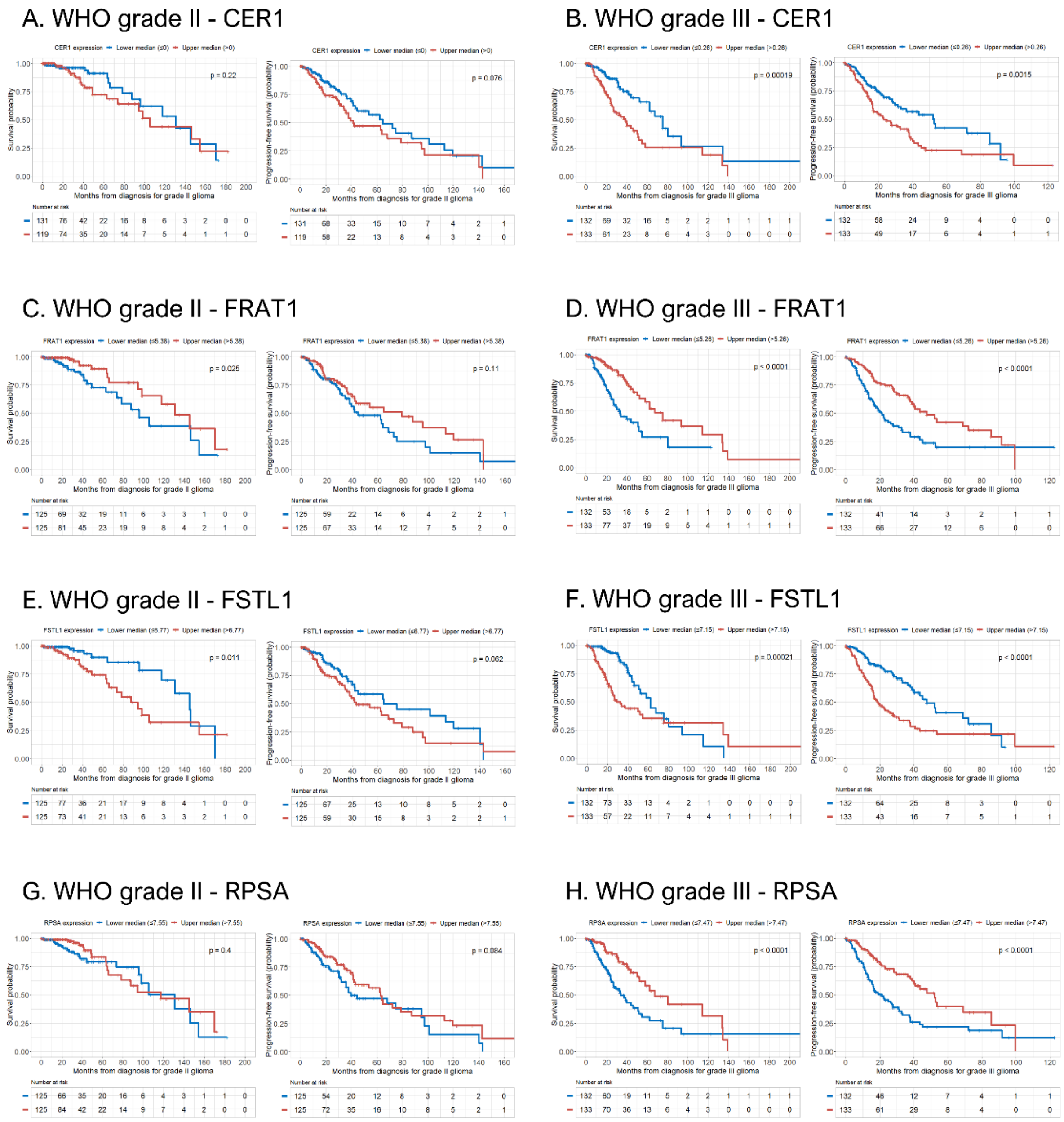

Supplementary Figure S4. Kaplan–Meier curves showing OS and PFS rates for the selected four significant genes related to Wnt/β-catenin signaling pathway in glioma patients classified by WHO grade.

OS, overall survival; PFS, progression-free survival; WHO, World Health Organization.

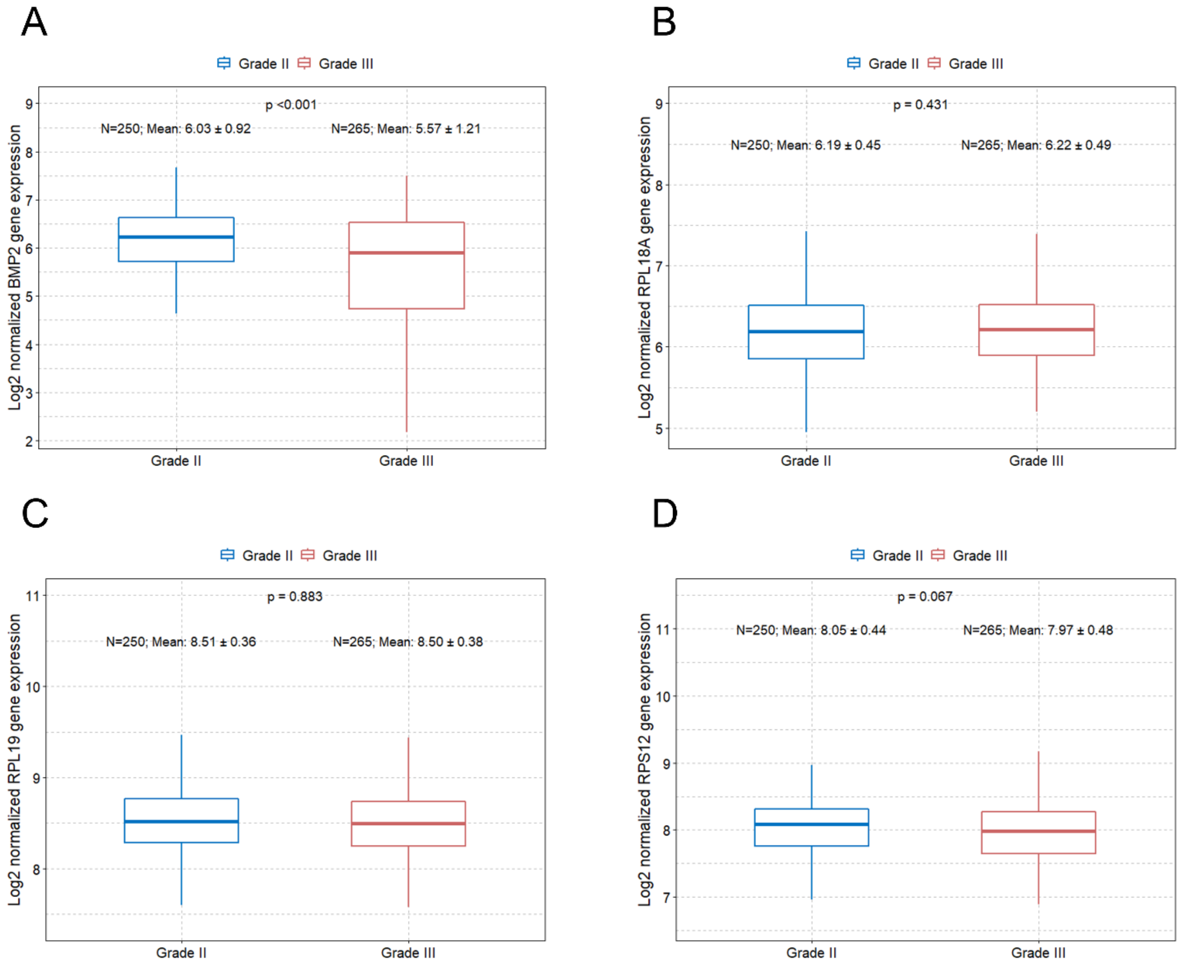

Supplementary Figure S5. Comparison of gene mRNA expressions between grade II and grade III glioma according to four additional significant genes: (A) *BMP2*; (B) *RPL18A*; (C) *RPL19*; and (D) *RPS12*.

BMP2, bone morphogenetic protein 2; *RPL18A*, ribosomal protein L18A; *RPL19*, ribosomal protein L19; *RPS12*, ribosomal protein S12.

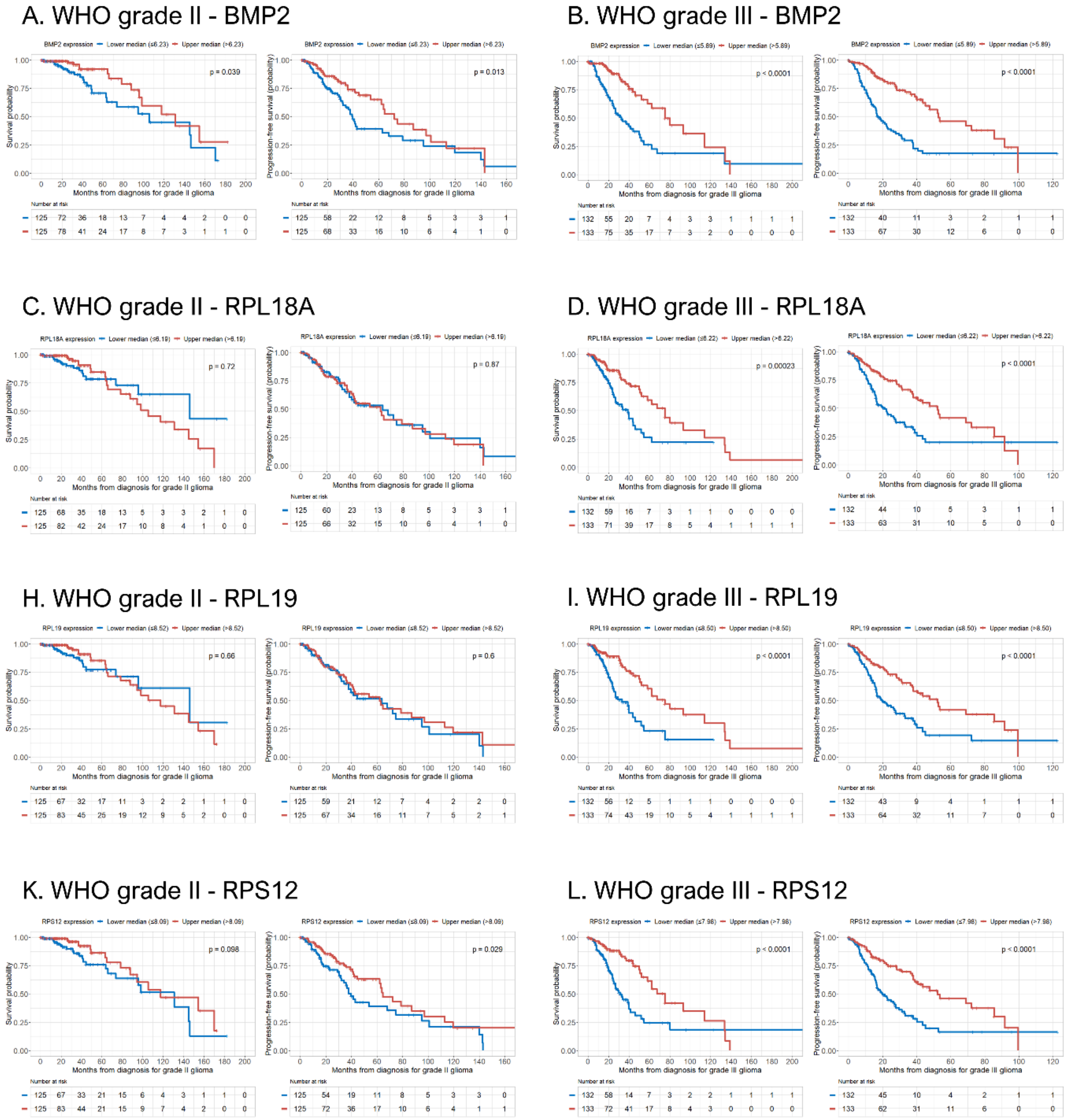

Supplementary Figure S6. Kaplan–Meier curves showing OS and PFS rates for additional four significant genes in glioma patients classified by WHO grade.

OS, overall survival; PFS, progression-free survival; WHO, World Health Organization.
